# Twelve-month service use, suicidality and mental health problems of European adolescents after a school-based screening for current suicidality

**DOI:** 10.1007/s00787-020-01681-7

**Published:** 2020-12-15

**Authors:** Michael Kaess, N. Schnyder, C. Michel, R. Brunner, V. Carli, M. Sarchiapone, C. W. Hoven, C. Wasserman, A. Apter, J. Balazs, J. Bobes, D. Cosman, C. Haring, J.-P. Kahn, H. Keeley, A. Kereszteny, T. Podlogar, V. Postuvan, A. Varnik, F. Resch, D. Wasserman

**Affiliations:** 1 University Hospital of Child and Adolescent Psychiatry and Psychotherapy, University of Bern, Bolligenstrasse 111, Stöckli, 3000 Bern 60, Switzerland; 2 Clinic of Child and Adolescent Psychiatry, Psychosomatics and Psychotherapy, University of Regensburg, Regensburg, Germany; 3 School of Public Health, The University of Queensland, Brisbane, Australia; 4 Policy and Epidemiology Group, Queensland Centre for Mental Health Research, Brisbane, Australia; 5 Department of Learning, Informatics , Management and Ethics, National Swedish Prevention of Mental Ill-Health and Suicide (NASP)/WHO Collaborating Centre for Research, Methods Development and Training in Suicide Prevention, Karolinska Institute, Stockholm, Sweden; 6 Department of Medicine and Health Science, University of Molise, Campobasso, Italy; 7 National Institute for Health, Migration and Poverty, Rome, Italy; 8 Division of Child and Adolescent Psychiatry, New York State Psychiatric Institute, Columbia University, New York, USA; 9 Department of Psychiatry, New York State Psychiatric Institute, Columbia University, New York, USA; 10 Department of Epidemiology, Mailman School of Public Health, Columbia University, New York, USA; 11 Feinberg Child Study Centre, Schneider Children’s Medical Centre, Tel Aviv University, Tel Aviv, Israel; 12 Bjørknes University College, Oslo, Norway; 13 Institute of Psychology, Eötvös Loránd University, Budapest, Hungary; 14 Department of Psychiatry, Centro de Investigación Biomédica en Red de Salud Mental, University of Oviedo, Oviedo, Spain; 15 Clinical Psychology Department, Iuliu Hatieganu University of Medicine and Pharmacy, Cluj-Napoca, Romania; 16 Institute for Clinical Evaluation, Department for Psychiatry and Psychotherapy B, State Hospital Hall, Tyrol, Austria; 17 Department of Psychiatry and Clinical Psychology, CHRU de Nancy and Centre Psychothérapique de Nancy, Université de Lorraine, Nancy, France; 18 North Cork Child and Adolescent Services, Health Service Executive South, Mallow Primary Healthcare Service, Gouldshill, Mallow Co Cork, Ireland; 19 Slovene Center for Suicide Research, Andrej Marusic Institute, University of Primorska, Koper, Slovenia; 20 Estonian-Swedish Mental Health and Suicidology Institute, Tallinn, Estonia; 21 Tallinn University School of Natural Science and Health, Tallinn, Estonia

**Keywords:** Suicide, Suicidal behaviour, Adolescents, Screening, Help-seeking, SEYLE

## Abstract

Suicide is one of the leading causes of death in adolescents and help-seeking behaviour for suicidal behaviour is low. School-based screenings can identify adolescents at risk for suicidal behaviour and might have the potential to facilitate service use and reduce suicidal behaviour. The aim of this study was to assess associations of a two-stage school-based screening with service use and suicidality in adolescents (aged 15 ± 0.9 years) from 11 European countries after one year. Students participating in the ‘Saving and Empowering Young Lives in Europe’ (SEYLE) study completed a self-report questionnaire including items on suicidal behaviour. Those screening positive for current suicidality (first screening stage) were invited to an interview with a mental health professional (second stage) who referred them for treatment, if necessary. At 12-month follow-up, students completed the same self-report questionnaire including questions on service use within the past year. Of the *N* = 12,395 SEYLE participants, 516 (4.2%) screened positive for current suicidality and were invited to the interview. Of these, 362 completed the 12-month follow-up with 136 (37.6%) self-selecting to attend the interview (screening completers). The majority of both screening completers (81.9%) and non-completers (91.6%) had not received professional treatment within one year, with completers being slightly more likely to receive it (*χ*^2^_(1)_ = 8.948, *V* = 0.157, p ≤ 0.01). Screening completion was associated with higher service use (OR 2.695, se 1.017, p ≤ 0.01) and lower suicidality at follow-up (OR 0.505, se 0.114, p ≤ 0.01) after controlling for potential confounders. This school-based screening offered limited evidence for the improvement of service use for suicidality. Similar future programmes might improve interview attendance rate and address adolescents’ barriers to care.

**Electronic supplementary material:**

The online version of this article (10.1007/s00787-020-01681-7) contains supplementary material, which is available to authorized users.

## Introduction

In Europe, suicide rates are on average the highest worldwide [1], with suicide being one of the leading causes of death in adolescents [2, 3]. Suicidal behaviour has serious consequences for the individual [4, 5], and negatively affects their families and friends [6]. Although mental healthcare is available in many European countries, the burden of both mental disorders and suicidal behaviour remains high. One potential reason is the low level of help-seeking behaviour within the mental healthcare system [7], which is most evident among youth [8]. Evidence suggests that only 20–40% of children and adolescents with mental health problems have been detected by health services, and only 25% received appropriate professional treatment [9]. Many adolescents that attempted suicide, reported earlier suicidal behaviour [10, 11] or engaged in deliberate self-harm [10, 12] but did not receive mental healthcare for it. Non-fatal self-harm and suicidal behaviour can precede suicide completion [13] but a progression might be prevented by timely intervention [14].

School-based screening interventions are considered useful for identifying adolescents at risk for suicidal behaviour [15, 16] and have the potential to facilitate service use [7]. School-based screening interventions typically involve a two-stage process [15, 17]. First, all students complete a brief self-report instrument to detect those at risk. Second, a mental health professional interviews those at risk to identify individuals who require ongoing support and, if needed, refers them to a subsequent intervention [15]. Studies conducting school-based screenings varied substantially in their number (between 4 and 45%) of young people identified as at risk for suicidal behaviour [15, 18]. This large difference in prevalence rates might be due to methodological differences in the studies. Although screenings have been criticised for their potential for high false-positive rates, it has been demonstrated that school-based screenings following a so-called two-stage approach (screening with high sensitivity and low specificity in the first stage, and enhancing specificity by in-depth assessment in the second stage) are clinically valid and reliable [19, 20] and may detect potential at-risk adolescents not otherwise identified [21]. For two-stage screenings to be effective, they should find people who are at risk and, if needed, facilitate access to treatment. While the usefulness of screenings to identify people at risk has been studied, much less is known whether they can facilitate access to treatment. An earlier study suggested that the referral after a two-stage screening for suicidality can facilitate adolescents’ access to mental health services at follow-up [7]. Whether this finding from the USA can be translated to the experience of young at-risk Europeans is so far unknown. In addition, factors, such as age, sex, mental health problems and well-being [18, 22, 23], that are associated with service use and might confound associations between screening on subsequent service use, need to be considered when evaluating such screening procedures. Two-stage school-based screenings are one potential option for indicated prevention involving individuals with subclinical symptoms and aiming to improve their service use, when necessary. This potentially improved service use might be indirectly associated adolescents’ symptoms and well-being at a later time. To the best of our knowledge, this has not been studied so far in the context of a large, multinational school-based screening. Within the framework of the ‘Saving and Empowering Young Lives in Europe’ (SEYLE) study [24], a two-stage screening for current suicidality was implemented as an emergency procedure within a large sample of adolescents. All students at risk for recent suicidality were immediately contacted and, if not directly reached, contacted several times to be invited for a clinical interview. Almost 80% of all at-risk students were reached but only 37.6% accepted the invitation for a clinical interview with various reasons for refusal with fewer than 10% refusing because they were already in contact with services [18]. We addressed the following research questions by conducting a 12-month follow-up of those who screened positive for current baseline suicidality: (1) how frequent was service use within 1 year among adolescents that completed the screening and those that did not and what type of services were used? (2) Is screening completion associated with follow-up service use controlled for baseline mental health problems and demographic characteristics (potential confounders)? (3) Do mental health problem differ between baseline and 12-month follow-up in the total sample, screening completers and non-completers, and service users and non-users? (4) Is screening completion associated with follow-up mental health problems when adjusting for service use and baseline mental health problems (potential confounders)?

## Methods

### Study design

The original SEYLE study is a randomised controlled trial (RCT) of the three school-based interventions and a minimal intervention/control group aiming at the primary prevention of suicidal behaviours [registered at the US National Institute of Health (NIH) clinical trial registry (NCT00906620), and the German Clinical Trials Register (DRKS00000214)]. Details on methodology and interventions have been described elsewhere [24, 25]. Eleven countries including Austria, Estonia, Germany, France, Hungary, Ireland, Israel, Italy, Romania, Slovenia, and Spain implemented the SEYLE study, with Sweden as the coordinating site. Local ethical committees granted approval to each study site. The countries were selected to provide a broad geographical representation of Europe. Researchers in each country randomly selected mixed-gender post-primary schools within a pre-determined and representative study site. A total of 264 schools were approached for participation, of which 179 schools accepted, with an overall response rate of 67.8%. The methodology of assessments and interventions were robust and homogenous across countries.

At baseline and at a 12-month follow-up, all students of the SEYLE study completed a self-report questionnaire in a school-based setting on, among other topics, sociodemographic characteristics, well-being, strengths and difficulties, depressive symptoms, and suicidal behaviour. Baseline data were assessed between November 2009 and December 2010, data for the follow-up 12 months later. To facilitate assessment of the change of these variables from baseline to follow-up, the same instruments were used. The questionnaire was adapted for adolescents. All used instruments were chosen by the SEYLE Consortium, have been validated and well-studied [24]. Students and their parents were informed about all procedures of the study and all gave written consent. One part of the baseline assessment was an emergency screening for current suicidality. This screening was performed before random allocation to the intervention arms was made; it aimed to identify adolescents at risk and offer them immediate support and referral if needed. The scope of this study focusses on the emergency screening for students that screened positive for current suicidality at baseline and completed the questionnaire at the 12-month follow-up.

### Screening for current suicidality and screening completion

The screening followed above outlined two-stage approach. Two questions of the Paykel Suicide Scale (PSS) [26] were used to identify students with current (past 2 weeks) suicidality. Students that answered ‘yes’ to (a) ‘Have you tried to take your own life during the past 2 weeks?’ and/or students that answered ‘sometimes’, ‘often’, ‘very often’ or ‘always’ to (b) ‘During the past 2 weeks, have you reached the point where you seriously considered taking your life or perhaps made plans how you would go about doing it?’ were considered to be at risk for suicidality. These students were offered a clinical interview with a mental health professional and referred to subsequent services, if necessary (details on referral process in supplementary eMaterial 1). All students participating in the SEYLE study were included in the “emergency procedure”, i.e. completed the screening for current suicidality and subsequent interview procedure if applicable, before the school-based interventions were implemented. To avoid any stigmatisation, all students (including those screened positive for current suicidality) further continued the school-based intervention arm they were originally randomised to, but were excluded from the evaluation of the effectiveness of those interventions in the main effect paper of the SEYLE study [19]. Our variable screening completion (yes/no) indicates whether a student participated in both stages of the screening or not. This measure was used as independent variable in the regression analyses.

Across all countries, the screening process and the contents of the interview were standardised and performed according to the study protocol. However, depending on local regulations and resources, follow-up process and interview setting could vary. For example, in some centres, the interview took place in schools, while in others, it took place at a local mental health facility. In most countries, both at-risk students and their parents were contacted via phone to schedule the clinical interview (see supplement 1 [18] on arrangement of interview).

### Measures for mental health problems and well-being

We assessed current (past 2 weeks) suicidality with a modified version of the 5-item PSS [26] including five different severity levels of suicidal ideation and behaviour (feeling that life is not worth living, wishing for death, thoughts of suicide without intent, seriously considering or planning suicide, and having attempted suicide). All but the question about suicide attempt (yes/no) was rated on a 6-point Likert scale ranging from ‘never’ to ‘always’. Cronbach’s alpha for this measure (*α* = 0.79) was acceptable.

We assessed depressive symptoms in the past 2 weeks with 20-items of the 21-item Beck’s Depression Inventory (BDI-II) [27], excluding the item ‘loss of libido’, since it was considered inappropriate for adolescents in some cultural settings [28]. Students rated the items on a 4-point Likert scale and we computed sum scores for further analyses with higher values representing more depressive symptoms. Cronbach’s alpha (*α* = 0.86) was good.

We assessed past 6 months difficulties with four of the five subscales of the Strengths and Difficulties Questionnaire (SDQ) [29]. Subscales emotional symptoms, conduct problems, peer relation problems, and hyperactivity and/or inattention contain five items each and are rated on a 3-point Likert scale. A total difficulty score is generated by summing up scores from these four subscales with higher values indicating more difficulties. Cronbach’s alpha for this measure (*α* = 0.74) was acceptable.

We assessed positive mood, vitality, and general interest during the past 2 weeks with the 5-item WHO Well-being Scale (WHO-5) [30] which is reliable in adolescents samples [31]. Items are rated on a 5-point Likert scale and we generated sum scores with higher values representing better well-being. The Cronbach’s alpha (*α* = 0.80) was good.

### Service use

We asked students at the 12-month follow-up which type of service and support they had received since the implementation of the SEYLE study. Possible answer categories were: medication, professional one-on-one therapy, group therapy, advice from a health professional, healthy lifestyle group, a mentor to talk to, and others. Since we were interested in service use from health professionals, we created the binary variable of ‘service use’ with the answers ‘yes’ if students received medication, professional one-on-one therapy, group therapy, or advice from a health professional and ‘no’ they received other or no care.

### Statistical analyses

Inclusion criteria for data analyses of the current study were: at-risk for current suicidality at baseline and completion of the 12-month follow-up self-report questionnaire. We analysed differences in descriptive data at baseline between screening completers and non-completers. We analysed differences in depressive symptoms, suicidality, difficulties, and well-being from baseline to follow-up for the total sample, screening completers and non-completers, and service users and non-users. If variables did not met assumptions for *t* test, Mann–Whitney *U* test and Wilcoxon signed-rank tests were used; if they did, independent and paired *t* tests were used. To control for potential confounders, the associations between screening completion and follow-up service use were modelled with simultaneous logistic regressions adjusted for age, sex, intervention group, and baseline mental health problems; the associations between screening completion and follow-up mental health problems were modelled with simultaneous linear regressions for continuous and simultaneous ordered logistic regressions for ordered dependent variables, adjusted for service use, intervention group and baseline mental health problems. In accordance with STROBE guidelines [32], we report unadjusted and adjusted regression models. Missing data (0.6–8.3% per variable) were listwise deleted. The statistical analyses were done in Stata version 15 (Stata Corporation, College Station, TX, USA).

## Results

### Sample

**Fig. 1:**
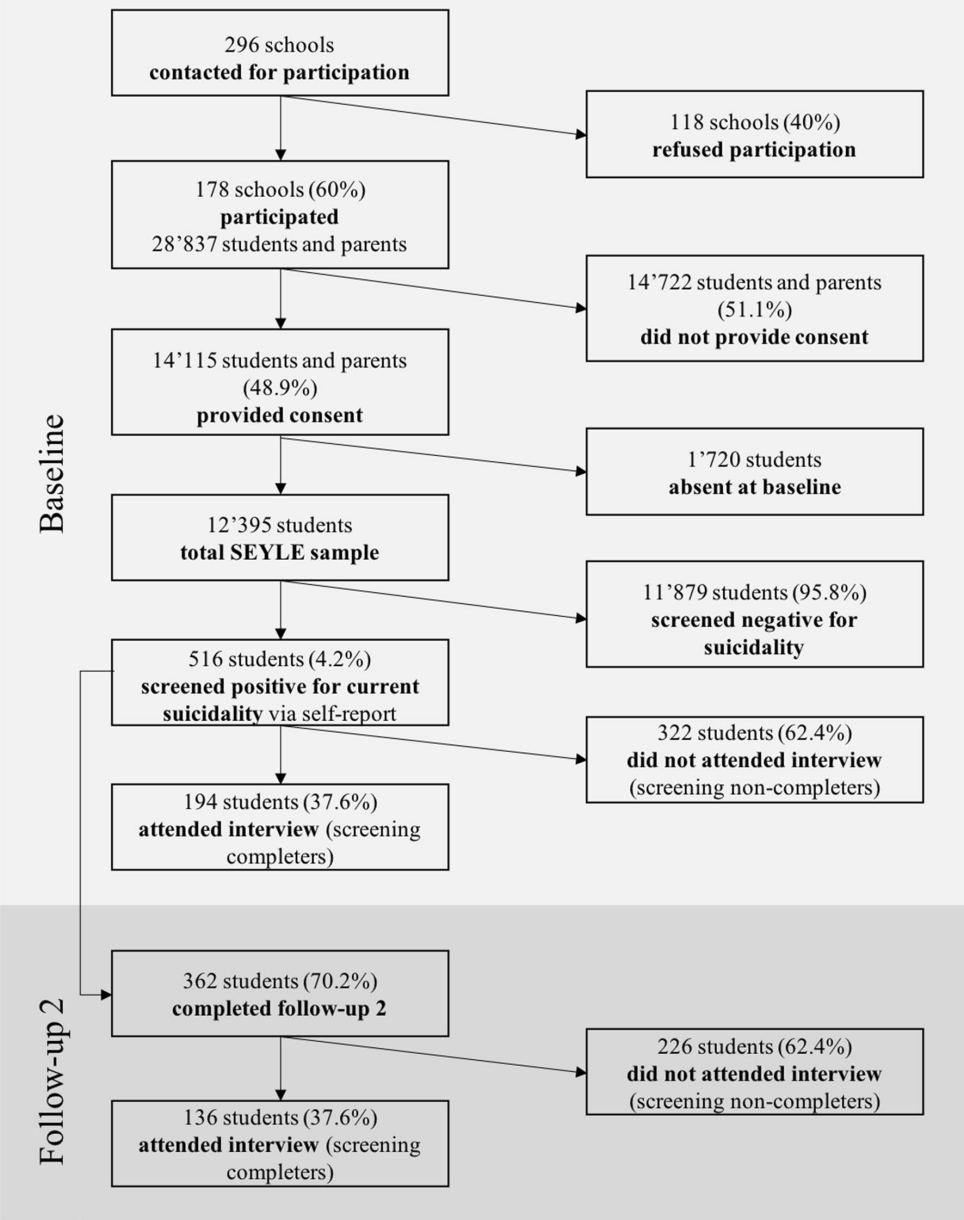
Flow-chart of recruitment and participation of students in SEYLE study, participation on screening process at baseline (11/2009–12/2010) and completion of follow-up questionnaire (12 months after baseline)

A total of *N* = 12,395 school-based adolescents participated in the SEYLE study. Of these, 516 (4.2%) students screened positive for current suicidality via self-report at baseline and 194 (37.6%) attended the interview (screening completers). Most students who did not attend the interview (non-completers) were unwilling to do so (58.1%; see [18]). The 12-month follow-up self-report was completed by 362 students. Of these, 136 students (37.6%) were screening completers (Fig. 1).

Subsequent data analyses and results refer to the 362 students that were considered to be at risk for current suicidality at baseline and completed the 12-month follow-up questionnaire (hereafter, completers). eTable 1 reports baseline sample characteristics. Completers and non-completers showed no differences in most variables, with the only exception that completers had significantly higher scores of depressive symptoms at baseline.

### Follow-up service use and type of services used

The majority (87.6%) of students that were at risk for current suicidality at baseline did not engage in treatment with a health professional within 1 year with slightly more screening completers than non-completers engaging in it (Table 1). Regardless of completion or non-completion, most at-risk adolescents that used services with a health professional were engaged in professional one-to-one therapy, followed by having received counsel from a health professional. Only few at-risk adolescents received medication (Table 1). Among screening completers, service use did differ between students that were referred to a subsequent treatment and students that were not (Table 1).

**Table 1:** Follow-up service use in total sample, among screening completers and those referred

	Follow-up service use^a^ in total sample (*N* = 362)		
	Yes	No	Statistics *χ*^2^_(df)_, *p*, Cramer’s *V*^b^
Screening completion, *n* (%)			
Yes No Total	26 (19.12)^c^ 19 (8.41)^c^ 45 (12.43)	110 (80.88)^c^ 207 (91.59)^c^ 317 (87.57)	*χ*^2^_(1)_ = 8.948, *p* ≤ 0.01, *V* = 0.157

	Screening completers	Screening non-completers	
Type of service used, *n* (%)			
Medication Professional one-to-one therapy Group therapy Advice from health professional Not professional treatment Total	1 (2.70)^c^ 18 (48.65) 1 (2.70) 6 (16.22) 11 (29.73) 37 (61.71)	4 (18.18)^c^ 9 (40.91) 0 (0) 6 (27.27) 3 (13.64) 22 (37.29)	*χ*^2^_(4)_ = 7.011, *p* = 0.135, *V* = 0.345

	Follow-up service use^a^ among screening completers (*n* = 136)		
	Yes	No	Statistics *χ*^2^_(df)_, *p*, Cramer’s *V*^b^
Referral, *n* (%)			
Yes No Total	18 (25.35) 8 (12.31) 26 (19.12)	53 (74.65) 57 (87.69) 110 (80.88)	*χ*^2^_(1)_ = 3.734, *p* = 0.053, *V* = 0.166

‘*χ*^2^_(df)_’ Chi-squared test with degrees of freedom

^a^Service use refers to professional service use without category ‘not professional treatment’

^b^Cramer’s *V* of 0.1, 0.3, and 0.5 represent small, medium, and large effect size, respectively

^c^Number in cells larger/smaller than expected

### Associations of screening completion with 12-month follow-up service use adjusted for potential confounders

After controlling association between screening completion and service use for baseline symptoms, difficulties, well-being, sociodemographic variables and intervention group, screening completion was associated with higher odds of service use (Table 2; unadjusted models in eTable 2).

**Table 2:** Adjusted logistic regression of variables associated with service use within 1 year (*n* = 326)

	Service use within 1 year
	OR (se)
Screening completion^a^	2.695** (1.017)
Baseline depressive symptoms^b^	1.046* (0.022)
Baseline suicidality^b^	0.234 (0.188)
Baseline WHO well-being^b^	0.995 (0.011)
Baseline difficulties^b^	1.056 (0.044)
Age^b^	1.536* (0.326)
Sex^c^	0.921 (0.367)
Intervention group^d^	
Question, persuade, and refer Youth aware of mental health programme Screening by professionals	0.470 (0.245) 0.932 (0.454) 0.478 (0.247)

**p ≤ 0.01, *p ≤ 0.05; *se* standard error; *R*^2^ = 0.152

^a^Reference category: no

^b^Reference: less depressive symptoms, lower suicidality, well-being and difficulties, and younger age, respectively

^c^Reference category: male

^d^Reference category: control group

### Differences in mental health problems and well-being between baseline and 12-month follow-up

In the total sample, in both screening completers and non-completers, and in both service users and non-users, depressive symptoms, suicidality, and difficulties significantly decreased, while well-being significantly increased, between baseline and 12-month follow-up (eTable 3). Regardless whether the total sample, completers or service users are examined, effect sizes indicate that the strongest decrease was for suicidality and depressive symptoms. Service users generally reported more symptoms and difficulties, and lower well-being both at baseline and follow-up than non-users. In particular, service users reported higher levels of suicidality at follow-up than non-users (eTable 3).

### Association of screening completion with 12-month follow-up mental health problems adjusted for potential confounders

After controlling association between screening completion and follow-up mental health problems and well-being for baseline symptoms, difficulties, well-being, service use, and intervention group, screening completion was associated with lower depressive symptoms, lower suicidality, less difficulties, and better well-being at 12-month follow-up (Table 3; unadjusted models in eTable 4).

**Table 3:** Adjusted linear or ordered logistic regression models of variables associated with 12-month follow-up symptoms, difficulties, and well-being

	Depressive symptoms^d^	Suicidality^e^	Well-being^d^	Difficulties^d^
	*β* (se)	OR (se)	*β* (se)	*β* (se)
Screening completion^a^	− 3.535** (1.233)	0.505** (0.114)	7.870** (2.598)	− 0.324 (0.598)
Service use^b^	5.073** (1.821)	1.879 (0.610)	− 5.326 (3.861)	0.774 (0.896)
Intervention group^c^				
Question, persuade, and refer Youth aware of mental health programme Screening by professionals	− 0.778 (1.619) − 2.222 (1.627) 1.200 (1.618)	0.861 (0.248) 0.839 (0.247) 0.752 (0.223)	− 0.156 (3.461) − 1.120 (3.447) 1.359 (3.432)	− 0.182 (0.789) 0.232 (0.785) 0.376 (0.786)
Baseline depressive symptoms^d^	0.297*** (0.064)	1.045*** (0.012)	− 0.003 (0.138)	0.069* (0.031)
Baseline suicidality^e^	0.510 (1.893)	1.199 (0.399)	5.768 (3.980)	0.350 (0.915)
Baseline well-being WHO^d^	− 0.014 (0.032)	1.006 (0.006)	0.222** (0.068)	0.003 (0.016)
Baseline difficulties^d^	0.205 (0.133)	1.027 (0.025)	− 0.374 (0.284)	0.412*** (0.064)

Depressive symptoms *R*^2^ = 0.208; suicidality pseudo *R*^2^ = 0.040; well-being *R*^2^ = 0.107; difficulties *R*^2^ = 0.240

***p ≤ 0.001; **p ≤ 0.01; *p ≤ 0.05

*OR* odds ratio, *β* regression coefficient, *se* standard error

^a^Reference category: screening not completed

^b^Reference category: no service use

^c^Reference category: control group

^d^Reference: lower depressive symptoms, difficulties, well-being

^e^Reference category: seriously considered suicide

## Discussion

This study had four key findings. First, both for screening completers and non-completers, 1-year service use rates of adolescents that were at risk for current suicidality at baseline were concerningly low with the majority (> 85%) not using any professional help. Second, adolescents that completed the screening were slightly more likely to engage in professional treatment even after controlling for baseline mental health problems and well-being, age, sex, and intervention group. Third, among adolescents with current suicidality at baseline, mental health problems and suicidality generally decreased while well-being increased from baseline to 12-month follow-up. Fourth, among screening completers, mental health problems and suicidality decreased and well-being increased more than among non-completers. This association was controlled for baseline mental health problems, suicidality and well-being, and for service use and intervention group.

The findings of this study provide us with a picture of the possible potential of a two-stage screening approach for current suicidality regarding service use with a health professional and regarding suicidality, depressive symptoms, difficulties, and well-being after 1 year. However, it also outlines potential room for improvement and limitations. Generally, the SEYLE study is so far the largest RCT involving school-aged adolescents aimed at suicide prevention for this target group. It has high response rates and good follow-up rates and includes a suicide screening that is both sensitive and specific. We looked at associations of suicide screening on later service use and on adolescents’ mental health problems for the first time in a European sample presented with current suicidality. Despite these strengths, limitations of the current study have to be considered. For ethical reasons, all adolescents that were at risk for current suicidality at baseline were offered the immediate screening intervention. Furthermore, screening completion was self-selected by adolescents. For these two reasons, results of the current study are not based on a RCT and do not allow causal conclusions. However, we do also not expect that the RCT design of the original study had any effect on our results as the referral process was done before the school-based interventions were implemented and because we have statistically controlled for potential effects of the intervention arms. Furthermore, screening completers might have been more motivated to seek professional help even before completing the screening. Following this hypothesis, the observed association between screening completion and higher frequency of service use could have been influenced by the higher baseline symptoms and difficulties of the completer group potentially underlying the stronger motivation for treatment. All involved countries performed the standardised screening process including an interview according to the study protocol. Several steps, such as contacting adolescents multiple times and contacting the parents, were taken to increase interview attendance rate but it was still low. However, some follow-up processes and interview settings varied slightly. For example, study locations that used schools as interview settings had higher interview attendance rates than those that used the study centre and/or the local mental health institution [18]. Future studies with a similar design might consider offering the interviews with the mental healthcare professionals at schools and increasing mental health awareness among adolescents; this might lead to a better attendance rate. We were not able to account for different healthcare systems between countries or their coverage of mental healthcare. Because of these two points, we are not able to draw conclusions about adolescents from specific countries, but only about European adolescents in general. However, we conducted sensitivity analyses entering country as a covariate in our regression models but did not find significant country differences with regard to help-seeking. While the relatively small groups of completers for each country do not allow ruling out small country effects, these analyses indicate that our results may be generalized to the overall European population. Last, we only focussed on adolescents’ perspectives, without addressing the influence their parents’ perspectives might have. In addition to adolescents themselves, parents are important stakeholders in adolescent mental health care; but parents and adolescents may report different mental health concerns in relation to the adolescent or disagree whether or what type of mental health care is perceived as being needed [33, 34]. Future studies might include both parents’ and adolescents’ perspectives in relation to mental health problems and service use.

Our findings suggest that despite the positive association between completion of screening and service use with a health professional described before [7], service use rates of adolescents with current suicidality remain low. A lack of perceived need for care or other barriers to care [35, 36] including stigma [37, 38] are some of the reasons why people often do not use treatment. The low explained variance of our model indicates that other factors are involved in service use than the one we have focussed on. Most at-risk adolescents that engaged in services within 1 year received professional one-to-one therapy while only a few at-risk adolescents received medication. It is difficult to judge if the received services were appropriate because we cannot determine which symptoms, problems, and disorders were treated.

Furthermore, our findings suggest that for all adolescents with current suicidality at baseline, mental health problems decreased and well-being increased from baseline to follow-up, despite the fact that many did not receive professional mental health care. This might seem contradictory at first, however, it should be noted that all participants received one of the SEYLE interventions after completing the screening, which may have contributed to the overall decline in mental health problems. In addition, it has been previously reported that among people with a depressive, anxiety and substance use disorder that had never been treated, remission rates were approximately 50% without subsequent treatment [39]. Depressive symptoms and suicidality decreased more and well-being increased more for completers than non-completers. While this may be attributed to increased service use, there is still the possibility that the general motivation for change and help-seeking itself may have an impact on mental health symptoms trajectories. Our finding can, again, be compared to another finding of the same earlier study that showed that despite remission of symptoms in both groups that did or did not access services, participants that did not access services had a lower quality-of-life score than those that did access services [39].

Completers and non-completers had similar difficulties including emotional symptoms, conduct problems, hyperactivity and/or inattention, and peer relationship problems. The improvement of depressive symptoms, suicidality, and well-being might not translate to other problems that adolescents might experience, such as conduct and peer relationship problems. Furthermore, depressive symptoms, suicidality, and well-being relate to the past 2 weeks, while difficulties relate to the past 6 months. The positive association of screening completion and adolescents’ mental health might only occur after a certain amount of time has passed.

School-based screening programs might be useful tools to detect adolescents at risk for current suicidality. Facilitating service use rates seems to be more difficult because the overall level of help-seeking among suicidal adolescents remained low, even after screening completion and subsequent referral. Future school-based screening studies might conduct interviews at schools to improve attendance rate and address adolescents’ barriers to care.

## Electronic supplementary material

Below is the link to the electronic supplementary material.

Supplementary file1 (DOCX 36 kb)

## Data Availability

The authors have had complete access to the raw study data and the access is on-going. The corresponding author can be contacted if access to the data should be desired.
