# Supplementary material for: Twelve-month service use, suicidality and mental health problems of European adolescents after a school-based screening for current suicidality
Source: Eur Child Adolesc Psychiatry. 2020 Dec 15;31(2):229–38. doi: 10.1007/s00787-020-01681-7 (PMC8837507; doi:10.1007/s00787-020-01681-7)
Supplement: Supplementary file 1 — Supplementary file1 (DOCX 36 kb) [file 787_2020_1681_MOESM1_ESM.docx]

**Supplement to:**

***‘*Longitudinal effect of school-based screening for current suicidality on follow-up service use, suicidality and mental problems among European adolescents’**

Kaess, M.,^1,2*^ Schnyder, N.,^1,3,4*^ Michel, C.,^1^ Brunner, R.,^2^ Carli, V.,^5^ Sarchiapone, M.,^6,7^ Hoven, C. W.,^8,9,10^ Wasserman, C.,^6,8^ Apter, A.,^11^ Balazs, J.,^12,13^ Bobes, J.,^14^ Cosman, D.,^15^ Haring, C.,^16^ Kahn, J.-P.,^17^ Keeley, H.,^18^ Kereszteny, A.,^13^ Podlogar T.,^19^ Postuvan, V.,^19^ Varnik, A.,^20,21^ Resch, F.,^2^ Wasserman, D.^5,8^

^*^ indicates shared first authorship

^1^ University Hospital of Child and Adolescent Psychiatry and Psychotherapy, University of Bern, Bern, Switzerland;

^2^ Clinic of Child and Adolescent Psychiatry, Centre of Psychosocial Medicine, University of Heidelberg, Heidelberg, Germany;

^3^ School of Public Health, The University of Queensland, Brisbane, Australia;

^4^ Policy and Epidemiology Group, Queensland Centre for Mental Health Research, Brisbane, Australia;

^5^ Department of Public Health Sciences, National Swedish Prevention of Mental Ill-Health and Suicide (NASP)/WHO Collaborating Centre for Research, Methods Development and Training in Suicide Prevention, Karolinska Institute, Stockholm, Sweden;

^6^ Department of Medicine and Health Science, University of Molise, Campobasso, Italy;

^7^ National Institute for Health, Migration and Poverty, Rome, Italy;

^8^ Division of Child and Adolescent Psychiatry, New York State Psychiatric Institute, Columbia University, New York, USA;

^9^ Department of Psychiatry, New York State Psychiatric Institute, Columbia University, New York, USA;

^10^ Department of Epidemiology, Mailman School of Public Health, Columbia University, New York, USA;

^11^ Feinberg Child Study Centre, Schneider Children’s Medical Centre, Tel Aviv University, Tel Aviv, Israel;

^12^ Vadaskert Child and Adolescent Psychiatric Hospital, Budapest, Hungary;

^13^ Institute of Psychology, Eötvös Loránd University, Budapest, Hungary;

^14^ Department of Psychiatry, Centro de Investigación Biomédica en Red de Salud Mental, University of Oviedo, Oviedo, Spain;

^15^ Clinical Psychology Department, Iuliu Hatieganu University of Medicine and Pharmacy, Cluj-Napoca, Romania;

^16^ Institute for Clinical Evaluation, Department for Psychiatry and Psychotherapy B, State Hospital Hall, Tyrol, Austria;

^17^ Department of Psychiatry and Clinical Psychology, CHRU de Nancy and Centre Psychothérapique de Nancy, Université de Lorraine, Nancy, France;

^18^ National Suicide Research Foundation, Cork, Ireland;

^19^ Slovene Center for Suicide Research, Andrej Marusic Institute, University of Primorska, Koper, Slovenia;

^20^ Estonian-Swedish Mental Health and Suicidology Institute, Tallinn, Estonia;

^21^ Tallinn University School of Natural Science and Health, Tallinn, Estonia.

**Content:**

**eMaterial 1.** Guideline-based interview with mental healthcare professional of the SEYLE study to determine referral to a mental healthcare professional outside of the study.

**eTable 1.** Sociodemographic and clinical characteristics of sample at baseline with group comparisons of screening completers and non-completers.

**eTable 2.** Unadjusted and adjusted logistic regressions of associations with service use within one year.

**eTable 3.** Symptoms and problems at baseline and 12-month follow-up in the total sample, among sub-samples of screening completers and service users.

**eTable 4.** Unadjusted linear and ordered logistic regression models of associations with symptoms, difficulties, and well-being after one year.

**eMaterial 1.** Guideline-based interview with mental healthcare professional of the SEYLE study to determine referral to a mental healthcare professional outside of the study.

The questions bellow have to be understood as possible sample questions and may have been formulated differently by the respective examiner. Nevertheless, the examiner had to receive all necessary information from all young people at-risk for suicidal behaviour. All of the posed questions refer to a time period of the ***past six month***. The recommendation to see a mental healthcare professional was made when one of the questions or statements bellow was answered with ‘yes’. Even if there is enough information at the beginning of the interview to make a recommendation, the interview was completed. A referral was only made if the person was not already in contact with a mental healthcare professional.

1. Person has a BMI <16.5 or shows distinct overweight (BMI ≥30).
2. Person has used at least three times weight reducing strategies such as diet pills, throwing up, laxatives, excessive sports, skipped meals and/or showed clear body image disturbance.
3. Person shows extensive cigarette consumption (at least 10 cigarettes per day).
4. Person consumes alcohol on a regular basis (2-3 times per week, independent of amount).
5. Person had at least five episodes of drunkenness with signs of control loss.
6. Person shows first signs of an emotional of physical addiction to alcohol.
7. Person consumed at least three times illegal drugs that were not medically prescribed.
8. Person spends an average of at least four hours per day in front of the computer or game consoles beyond school assignments. Direct social contacts or necessary obligations are neglected and/or person comes into conflict with parents/legal guardians.
9. Person broke the law twice or engaged in at least two actions that endangered his/her own life or the life or another person.
10. Person had more than one unprotected sexual intercourse.
11. Person had sexual intercourse with more than three different partners.
12. Person is absent from classes without permission on a regular basis.
13. Person has been increasingly (almost every week) victim of bullying such as being kicked, pushed embarrassed etc.
14. Person does not spend time with friends after school time.
15. Person worries and fears a number of times a day or continuously for several hours.
16. Person feels irritated/annoyed every day or almost every day during at least half of the time or screams frequently, loses self-control.
17. Person suffers (almost) every day from unpredictable and quickly occurring erratic mood swings.
18. Person shows depressive symptoms such as abjection, joylessness, loss of interests etc. at least three times per week for more than three hours at a time.
19. Person shows depressive symptoms for a least two weeks in a row.
20. Person has already intentionally hurt him-/herself (cutting, burning, etc.) at least three times.
21. Person has occasionally concrete or emerging suicide thoughts.
22. Person has thoughts or wishes of death almost every week

**eTable 1.** Sociodemographic and clinical characteristics of sample at baseline with group comparisons of screening completers and non-completers

|  | Total sample (N=362) | Screening completers (n=136) | Screening non-completers  (n=226) | Statistics  χ^2^_(df)_, p, Cramer’s *V*^a^ /  U, p, Pearson’s *r^b^* / t_(df)_, p, Cohen’s *d*^c^ |
| --- | --- | --- | --- | --- |
| Sex, n (%)  *Female*  *Male* | 230 (63.5)  132 (36.5) | 91 (39.6)  45 (34.1) | 139 (60.4)  87 (65.9) | χ^2^_(1)_=1.072, p=0.301, *V*=0.054 |
| Age: mean±SD | 15±0.9 | 15±0.9 | 15±1 | t_(358)_=0.488, p=0.626, *d*=0.034 |
| Intervention group, n (%)  *Question, persuade, and refer*  *Youth aware of mental health programme*  *Screening by professionals*  *Controls* | 86 (23.8)  88 (24.3)  89 (24.6)  99 (27.4) | 32 (37.2)  28 (31.8)  38 (42.7)  38 (38.4) | 54 (62.8)  60 (68.2)  51 (57.3)  61 (61.6) | χ^2^_(3)_=2.271, p=0.518, *V*=0.0792 |
| PSS Suicidality, n (%)  *Seriously considered suicide*  *Suicide attempt* | 311 (85.9)  51 (14.1) | 115 (37.0)  21 (41.2) | 196 (63.0)  30 (58.8) | χ^2^_(1)_=0.329, p=0.566, *V*=0.030 |
| BDI depressiveness: mean±SD | 21 (21.5±11.9) | 24 (24.7±11.8) | 19 (19.6±11.6) | U=884325.68, p<0.001, *r*=0.203 |
| WHO well-being: mean±SD | 41.3±20.9 | 41.0±20.0 | 41.4±21.5 | t_(334)_=0.176, p=0.860, *d*=0.020 |
| Strength and difficulties questionnaire: mean±SD | 16.7±5.4 | 17.3±5.1 | 16.3±5.6 | t_(349)_=-1.663, p=0.097, *d*=-0.182 |

^a^ Cramer’s *V* of 0.1, 0.3, and 0.5 represent small, medium, and large effect size, respectively; ^b^ Pearson’s *r* of 0.1, 0.3, and 0.5 represent small, medium, and large effect size, respectively; ^c^ Cohen’s *d* of 0.2, 0.5, and 0.8 represent small, medium, & large effect sizes.

*Note* ‘p’ p-value; ‘χ^2^_(df)_’ Chi-squared test for categorical data with degrees of freedom; ‘U’ Mann-Whitney U-test for nonparametric data; ‘t_(df)_’ independent t-test with degrees of freedom.

**eTable 2.** unadjusted logistic regressions of associations with service use within one year.

|  | ***Service use after one year*** | |
| --- | --- | --- |
|  | *unadjusted* | |
|  | OR (95%-CI) | se |
| *Screening completion^a^* | 2.575** (1.364-4.860) | 0.575 |
| *Baseline depressive symptoms^b^* | 1.047** (1.020-1.075) | 0.014 |
| *Baseline suicidality^b^* | 0.560 (0.192-1.638) | 0.307 |
| *Baseline WHO well-being^b^* | 0.980* (0.963-0.996) | 0.008 |
| *Baseline difficulties^b^* | 1.111** (1.047-1.179) | 0.034 |
| *Age^b^* | 1.479* (1.049-2.085) | 0.259 |
| *Sex^c^* | 0.842 (0.445-1.596) | 0.275 |
| *Intervention group^d^*  Question, persuade, and refer  Youth aware of mental health programme  Screening by professionals | 0.532 (0.216-1.313)  0.899 (0.406-1.992)  0.512 (0.208-1.263) | 0.245  0.365  0.236 |

^a^ Reference category: no; ^b^ Reference: less depressive symptoms and lower suicidality,

well-being, difficulties, and age respectively; ^c^ Reference category: male; ^d^ Reference category: control group.

*note* ** p≤0.01, *p≤0.05; *OR* odds ratio; *se* standard error.

**eTable 3.** Symptoms and problems at baseline and 12-month follow-up in the total sample, among sub-samples of screening completers and service

users.

|  |  | *Screening completers(SC)* | *Screening non-completers(SNC)* | *SC* | *SNC* | | *Total sample* | |
| --- | --- | --- | --- | --- | --- | --- | --- | --- |
|  |  | Median(mean±SD) | | Statistics: Z^a^, p, *r*^b^ or t_(df)_^c^, p, *d*^d^ | |  | Median(mean±SD) | Statistics: Z^a^, p, *r*^b^ or t_(df)_^c^, p, *d*^d^ |
| BDI | Baseline  Follow-up | 24(24.70±11.76)  9.5(11.55±10.27) | 19(19.62±11.63)  8(12.51±12.12) | Z=8.370^***^  *r*=0.737 | Z=8.087^***^  *r*=0.554 | | 21(21.53±11.92)  9(12.15±11.46) | Z=11.701^***^  *r*=0.633 |
| PSS | Baseline  Follow-up | 4(4.15±0.36)  1(1.41±1.53) | 4(4.13±0.34)  1(1.78±1.79) | Z=9.928^***^  *r*=0.864 | Z=12.014^***^  *r*=0.810 | | 4(4.14±0.35)  1(1.64±1.71) | Z=15.647^***^  *r*=0.834 |
| WHO | Baseline  Follow-up | 44(41.01±20.02)  56(57.80±21.57) | 36(41.42±21.51)  52(51.67±24.58) | t_(126)_=-7.394^***^  *d*=0.656 | t_(201)_=-5.662^***^  *d*=0.398 | | 40(41.26±20.92)  52(54.02±23.62) | t_(328)_=-8.933^***^  *d*=0.492 |
| SDQ | Baseline  Follow-up | 17(17.29±5.13)  14(13.99±5.55) | 16(16.31±5.61)  13(13.50±5.81) | t_(132)_=6.597^***^  *d*=0.572 | t_(210)_=7.201^***^  *d*=0.496 | | 17(16.69±5.44)  13(13.69±5.71) | t_(343)_=9.742^***^  *d*=0.525 |
|  |  | *Service users(SU)* | *Non-service users(NSU)* | *SU* | *NSU* | |  |  |
|  |  | Median(mean±SD) | | Statistics: Z^a^, p, *r*^b^ or t_(df)_^c^, p, *d*^d^ | |  |  |  |
| BDI | Baseline  Follow-up | 28(27.33±11.59)  15(18.04±14.39) | 20(20.69±11.75)  8(11.27±10.71) | Z=3.761^***^  *r*=0.561 | Z=11.132^***^  *r*=0.646 | |  |  |
| PSS | Baseline  Follow-up | 4(4.09±0.29)  3(2.33±1.68) | 4(4.15±0.36)  1(1.54±1.70) | Z=5.131^***^  *r*=0.765 | Z=14.768^***^  *r*=0.843 | |  |  |
| WHO | Baseline  Follow-up | 32(33.83±19.49)  48(48.09±25.93) | 40(42.33±20.94)  56(54.58±23.20) | t_(41)_=-4.241^***^  *d*=0.654 | t_(286)_=-8.014^***^  *d*=0.473 | |  |  |
| SDQ | Baseline  Follow-up | 20(19.44±5.50)  17(15.84±5.70) | 16(16.30±5.33)  13(13.38±5.66) | t_(41)_=4.013^***^  *d*=0.619 | t_(301)_=8.883^***^  *d*=0.511 | |  |  |

^a^Wilcoxon signed-rank test; ^b^Pearson *r* with 0.1, 0.3, & 0.5 representing small, medium, & large effect sizes; ^c^Paired t-test; ^d^Cohen’s *d* with 0.2, 0.5, & 0.8 representing small, medium, & large effect sizes.

*Note* ^***^p≤0.001; BDI: Becks Depression Inventory; PSS: Paykel Suicide Scale; WHO: WHO Well-Being Index; SDQ: Strengths and Difficulties Questionnaire.

**eTable 4.** unadjusted linear and ordered logistic regression models of associations with symptoms, difficulties, and well-being after one year.

|  | ***Depressiveness^d^ after 1 year*** | | ***Suicidality^e^***  ***after 1 year*** | | ***Well-being^d^***  ***after 1 year*** | | ***Difficulties^d^***  ***after 1 year*** | |
| --- | --- | --- | --- | --- | --- | --- | --- | --- |
|  | *unadjusted* | | *unadjusted* | | *unadjusted* | | *unadjusted* | |
|  | β | se | OR | se | β | se | β | se |
| *Screening completion^a^* | -0.965 | 1.272 | 0.721 | 0.144 | 6.126* | 2.545 | 0.493 | 0.626 |
| *Service use^b^* | 6.773*** | 1.797 | 2.305** | 0.651 | -6.782 | 3.796 | 2.460** | 0.912 |
| *Intervention group^c^*  Question, persuade, and refer  Youth aware of mental health programme  Screening by professionals | 0.221  -2.876  1.043 | 1.715  1.715  1.699 | 0.915  0.868  0.826 | 0.246  0.238  0.225 | 0.751  1.534  2.725 | 3.553  3.553  3.488 | -0.069  -0.868  -0.109 | 0.853  0.851  0.843 |
| *Baseline depressiveness^d^* | 0.390*** | 0.048 | 1.041*** | 0.009 | -0.280** | 0.106 | 0.176*** | 0.024 |
| *Baseline suicidality^e^* | -0.796 | 1.851 | 1.199 | 0.335 | 5.228 | 3.732 | 0.288 | 0.896 |
| *Baseline well-being WHO^d^* | -0.120*** | 0.030 | 0.991 | 0.005 | 0.254*** | 0.059 | -0.057*** | 0.015 |
| *Baseline difficulties^d^* | 0.604*** | 0.110 | 1.069*** | 0.020 | -0.656** | 0.226 | 0.475*** | 0.050 |

***p≤0.001; **p≤0.01; *p≤0.05; all estimates without star are not significant.

OR odds ratio; se standard error; β regression coefficient

^a^ Reference category: Screening not completed (no interview); ^b^ Reference category: no service use with health professional; ^c^ Reference category: control group; ^d^ Reference: lower depressive symptoms, difficulties, well-being; ^e^ Reference category: seriously considered suicide; ^e^ Reference category: no suicidal ideation.
